# Where Mental and Hormonal Health Meet: Improving Women’s Health Care in Psychiatric Inpatients

**DOI:** 10.1192/bjo.2026.11408

**Published:** 2026-06-30

**Authors:** Hannah Kelso, Grace Denton, Bridget Martin, Alexandra Thatcher, Sophie Hickling

**Affiliations:** Avon and Wiltshire Mental Health Partnership NHS Trust, Bristol, United Kingdom

## Abstract

**Aims:**

There is growing awareness of the complex bidirectional interactions between hormonal and mental health, with fluctuations across the menstrual cycle, perimenopause and menopause influencing the onset, severity and course of psychiatric illness. Despite this, reproductive health is often poorly assessed and documented in psychiatric inpatient settings. An initial audit across multiple inpatient wards demonstrated a substantial deficit in theidentification and documentation of reproductive health information. Our trust policy recommends that women are asked within 24 hours of admission about menstrual problems, menopause and breastfeeding; however, discussing these sensitive issues is often inappropriate or overlooked during acute psychiatric admissions. In this ongoing quality improvement project (QIP) we aim to increase awareness and detection of women’s health problems in female psychiatric inpatients using a trauma-informed approach led by trained nursing staff. We hypothesised that shifting responsibility from early medical clerking to appropriately timed nurse-led conversations would be more acceptable and feasible, while improving identification of clinically relevant issues.

**Methods:**

The QIP intervention was initiated on a single 15-bed female acute psychiatric ward. Nurses with additional training in women’s health were designated as Women’s Health Champions (WHCs). Their role included offering one-to-one conversations with service users when clinically appropriate, running twice-monthly educational groups, providing written information on the ward and escalating concerns to the medical team. The first Plan–Do–Study–Act (PDSA) cycle focused on assessing feasibility and uptake of one-to-one conversations over a three-week period, alongside qualitative identification of implementation challenges.

**Results:**

During the first QIP cycle, 7 of 15 service users were deemed well enough to participate in a one-to-one women’s health conversation. Three service users were offered a conversation over three weeks; two engaged in brief discussions. Barriers identified included limited nursing time, fluctuating mental state of service users and uncertainty around documentation and information-sharing. Key process issues to be addressed in future cycles: the need for thorough documentation to avoid missing ‘red flags’; obtaining explicit consentto share information with the medical team, and documenting whether a service user is well enough to participate.

**Conclusion:**

Early findings suggest that a nurse-led, trauma-informed model for addressing women’s health in psychiatric inpatients is feasible but constrained by acuity and resource limitations. While uptake was limited, the project identified clear, actionable system changes to support safer and more consistent practice. These results support revising existing policy to prioritise timing, consent and documentation rather than rigid early-admission targets.

